# The potential role of acrolein in plant ferroptosis-like cell death

**DOI:** 10.1371/journal.pone.0227278

**Published:** 2019-12-30

**Authors:** Péter Hajdinák, Ádám Czobor, András Szarka

**Affiliations:** Department of Applied Biotechnology and Food Science, Laboratory of Biochemistry and Molecular Biology, Budapest University of Technology and Economics, Budapest, Hungary; Institute for Sustainable Plant Protection, C.N.R., ITALY

## Abstract

The iron dependent, programmed cell death, ferroptosis was described first in tumour cells. It showed distinct features from the already known cell death forms such as apoptosis, necrosis and autophagy. The caspase independent cell death could be induced by the depletion of glutathione by erastin or by the inhibition of the lipid peroxide scavenger enzyme GPX4 by RSL3 and it was accompanied by the generation of lipid reactive oxygen species. Recently, ferroptosis-like cell death associated to glutathione depletion, lipid peroxidation and iron dependency could also be induced in plant cells by heat treatment. Unfortunately, the mediators and elements of the ferroptotic pathway have not been described yet. Our present results on *Arabidopsis thaliana* cell cultures suggest that acrolein, a lipid peroxide-derived reactive carbonyl species, is involved in plant ferroptosis-like cell death. The acrolein induced cell death could be mitigated by the known ferroptosis inhibitors such as Ferrostatin-1, Deferoxamine, α-Tocopherol, and glutathione. At the same time acrolein can be a mediator of ferroptosis-like cell death in plant cells since the known ferroptosis inducer RSL3 induced cell death could be mitigated by the acrolein scavenger carnosine. Finally, on the contrary to the caspase independent ferroptosis in human cells, we found that caspase-like activity can be involved in plant ferroptosis-like cell death.

## Introduction

Plants in their natural environments are exposed to a variety of biotic and abiotic stresses, including pathogens, drought, heavy metals, extreme temperature, salt and high light. Under these stress conditions, reactive oxygen species (ROS) derived from molecular oxygen can accumulate in plant cells [1–3]. Excess amount of ROS formation can lead to programmed cell death (PCD), moreover ROS are important factors in this process [4]. Several forms of cell death have been associated with excess ROS generation in animal cells [5]. None of them has been dedicated uniquely to ROS generation. However, recently ferroptosis, a new form of cell death was described that is iron dependent and exclusively caused by the accumulation of lipid-based peroxides [6]. Lipid peroxidation necessarily affect the ability of lipids to form functional membranes thus it can lead to the loss of membrane integrity and cell death [7]. Furthermore, the fragmentation of lipid alkoxyl radicals yields the production of reactive aldehydes such as malondialdehyde, 4-hydroxynonenal and acrolein [8]. These reactive aldehydes can diffuse from the site of lipid peroxidation to carbonylate proteins and induce cell death through the altered protein function [7]. The members of the aldo-keto reductase type 1C family (*AKR1C*) have the ability to detoxify toxic lipid metabolites produced by the oxidative lipid fragmentation during the execution of ferroptosis. Cells overexpressing the members of the *AKR1C* family showed resistance to the ferroptosis inducer erastin suggesting that these reactive aldehydes may play role in ferroptotic cell death [9]. Up to date solely two studies appeared on ferroptosis in plants [10,11]. The first report on plant ferroptosis found that heat stress triggered an iron-dependent cell death pathway that was similar to the ferroptosis in mammalian cells and could be characterized by depletion of GSH and accumulation of cytosolic and lipid ROS. This heat stress triggered, ferroptosis-like cell death (FCD) in plants could be suspended by the addition of the specific ferroptosis inhibitor Ferrostatin-1 or the membrane-permeable iron chelator ciclopirox olamine (CPX) [10]. These ferroptosis inhibitors could give protection only in moderate heat stress (55°C), however at higher temperature (77°C) they did not show any protective effect. As in mammalian cells, GSH plays key role in plant FCD, since GSH depletion was sufficient to trigger cell death in BSO (Buthionine sulfoximine) treated *Arabidopsis* roots. The lipid peroxide scavenging activity of GPX4 gives the background of the crucial role of GSH in ferroptosis in tumour cells [12]. Any effect that inhibit the activity or the substrate supply of the enzyme promotes ferroptosis.

In plants under environmental stress, the level of ROS including lipid peroxides in chloroplasts and mitochondria is increased [13]. As it was mentioned earlier different reactive carbonyl species such as malondialdehyde, 4-hydroxynonenal and acrolein were produced from these lipid peroxides by the catalysis with radical species or redox catalysts such as Fe^2+^ ion [13,14]. It was found that acrolein, one of the lipid peroxide derived reactive carbonyl species caused depletion of the GSH pool in BY-2 tobacco cells, then gradually lowered the ascorbate level and enhanced the ROS level finally caused cell death [15]. All these observations were substantially similar to the results found in the case of heat treatment induced FCD in *Arabidopsis thaliana*. Lipid peroxidation and GSH depletion are surely important factors in ferroptotic cell death [6,9,10]. However, the exact mediators of this cell death mechanism are still unknown. The similar conditions of acrolein generation raises the hypothesis that this molecule is a good candidate for the role of ferroptotic mediator. Thus, the effects of the known ferroptosis inductor RSL3 and acrolein were investigated and compared in this study to clarify the possible mediator role of acrolein in ferroptosis.

## Materials and methods

### Materials

Murashige and Skoog medium, 2,4-dichlorophenoxyacetic acid (2,4-D), kinetin, ethylenediaminetetraacetic acid (EDTA), triphenyl-tetrazolium chloride (TTC), 5-sulphosalicylic acid, 5,5-dithiobis(2-nitrobenzoic acid) (DTNB), deferoxamine, dimethyl sulfoxide (DMSO), acrolein, α-Tocopherol, Glutathione Reductase (GR), glutathione (GSH) and phenylmethylsulphonyl fluoride (PMSF) were obtained from Sigma-Aldrich. Ferrostatin-1, RSL3, Z-VAD-FMK and liproxstatin-1 were purchased from Selleckchem. 2’,7’-dichlorodihydrofluorescein diacetate (H_2_DCFDA) was purchased from Invitrogen. Ac-DEVD-AMC, Ac-DEVD-CHO and E64-d were from MedChemExpress. All other chemicals were from analytical or HPLC grade, and was purchased from Reanal, Hungary.

### *Arabidopsis thaliana* cell cultures

*Arabidopsis thaliana* suspension cells were cultivated as described earlier in Czobor *et al*. and Hajdinák *et al*. [2,16]. Briefly, cells were subcultured weekly by a tenfold dilution in fresh medium containing 0.44% MS + Gamborg; 3% Sucrose; 0.24 μg/ml 2,4-dichlorophenoxyacetic acid; 0.014 μg/ml Kinetin; 4 mM PBS (K_2_HPO_4_, KH_2_PO_4_); pH 5.8. The cells were grown in the dark at 22°C in a rotary shaker (120 rpm).

### Cell treatments

Ten ml of 4-day-old *Arabidopsis thaliana* cultures were pre-treated with different cell death inhibitors for 1 h, then the cells were treated with 400 μM of acrolein or 11.34 μM (5 μg/ml) of RSL3. The cell death inducers and inhibitors were dissolved in ethanol or DMSO. The concentration of ethanol or DMSO never reached the 0.1 (v/v) %.

### Determination of cell viability

Cell viability was determined by a slightly modified triphenyl-tetrazolium chloride (TTC) reduction assay [17]. Twenty mg TTC was dissolved in 1 ml of 50 mM sodium phosphate buffer (pH 7.5) for TTC stock solution. This stock solution was stored at 4°C in the dark. An aliquot of the cells was vacuum filtrated, weighted, and resuspended in 980 μl of 50 mM sodium phosphate buffer (pH 7.5). Twenty μl of TTC stock solution was added to the samples at a final concentration of 1.25 mM. The mixture was incubated in the dark for 1 h then it was centrifuged (16000 x *g*, 2 min). The supernatant was discarded, and the formazan salts were solubilized by the addition of 0.5 ml ethanol. After 2 h of incubation, the cells were centrifuged (16000 x *g*, 2 min), and the absorbance of the supernatant was measured at 485 nm. Cell viability was normalized to the wet weight of the cells.

### Determination of lipid peroxidation

Lipid peroxidation was determined according to the method of Hodges *et al*. and Poborilova *et al*. [18,19]. *Arabidopsis thaliana* cells were vacuum filtrated, washed with 50 mM sodium phosphate buffer (pH 7.5), and frozen by liquid nitrogen. The cells were resuspended (50 mg/ml) in ice cold 50 mM sodium phosphate buffer (pH 7.5) and homogenized by Potter–Elvehjem homogenizer for 20 s on ice. An aliquot of the cell homogenate was mixed with the same volume of “+ TBA” solution (20 (w/v) % trichloro acetic acid, 0.5 (w/v) % thiobarbituric acid (TBA), 0.01 (w/v) % butylated hydroxytoluene), and another aliquot with “-TBA” solution (20 (w/v) % trichloro acetic acid, 0.01% butylated hydroxytoluene). All these homogenates were mixed, and heat treated at 95°C for 30 min. The reaction was stopped by immediate cooling of the samples on ice. The cooled samples were centrifuged (3000 x *g*, 10 min, 4°C) and the absorbances of the supernatants were measured at 532 nm. The concentration of malonaldehyde equivalents was estimated using the standard curve (0–20 μM). The “-TBA” treated samples were used as blanks, and the results were normalized to the wet weight of the cells.

### Determination of glutathione

Glutathione content of the cells was measured by the DTNB (5,5′-Dithiobis(2-nitrobenzoic acid)) assay. A known amount of freshly vacuum filtrated *Arabidopsis thaliana* cells was homogenized in ice cold 5-sulphosalicylic acid (5 (w/v) %) by Potter–Elvehjem homogenizer. The homogenates were centrifuged (3000 x *g*, 10 min, 4°C), and the supernatants were diluted by assay buffer (45 mM Na_3_PO_4_, 0.45 mM EDTA, pH 7.5). Fifty μl of the diluted sample was added to 100 μl of reaction buffer (45 mM Na_3_PO_4_, 0.45 mM EDTA, 0.225 mM DTNB, 0.3 mM NADPH, 1.6 U/ml GR, pH 7.5) and mixed on a 96-well plate then the reaction was followed for 5 min at 405 nm (25°C). The initial 2 min of the linear phases were used to quantify the glutathione content. The results were normalized to the wet weight of the cells.

### Determination of ROS

175 μl of *Arabidopsis thaliana* cells was centrifuged (16000 x *g*, 2 min) and the supernatant was discarded. The pellet was resuspended in 350 μl 50 mM sodium phosphate buffer (pH 7.5) containing 10 μM H_2_DCFDA and the cells were incubated for 15 min in the dark then washed twice with 50 mM sodium phosphate buffer. Finally, the fluorescence was measured by a fluorescent plate reader (Varioskan LUX, excitation: 500 nm, emission: 540 nm). Samples without H_2_DCFDA were used as blanks.

### Determination of caspase-3-like protease activity

The activity of caspase-3-like protease (C3LP) was determined according to Biswas *et al*. and García-Heredia *et al*. [15,20].

*Arabidopsis thaliana* cells were harvested by vacuum filtration, frozen in liquid nitrogen and homogenized by Potter–Elvehjem homogenizer in 50 mM sodium acetate (pH 5.5), 50 mM NaCl, 1 mM EDTA, 1 mM PMSF and 0.1 mM E64-d. The homogenates were centrifuged (20000 x *g*, 15 min, 4°C) and the supernatants were collected. Fifty μl of the supernatant was added to 200 μl of assay buffer (20 mM sodium acetate (pH 5.5), 5 mM DTT, 0.1 mM EDTA and 1 mM PMSF), supplemented with 75 μM Ac-DEVD-CHO (a fluorogenic substrate of C3LP). The mixture was incubated at 37°C for 1 h, and the fluorescence was determined by a fluorescent plate reader (Varioskan LUX, excitation: 380 nm, emission: 445 nm).

For the C3LP inhibition assays, the samples were preincubated for 1 h at 37°C in the presence of 100 μM Ac-DEVD-CHO, a reversible inhibitor of C3LP [20]. The difference in the fluorescence intensities measured in the absence and in the presence of the inhibitor was considered as the activity of C3LP.

The results were normalized to the protein content of the sample that was determined by *Thermo Scientific*^*TM*^ *Pierce*^*TM*^ *BCA Protein Assay Kit*, according to the manufacturer’s instructions.

### Other methods

All data are expressed as means ± S.D. Statistical analyses were performed by *Student’s t test*.

## Results

### The cytotoxic effects of acrolein and RSL3 on *A. thaliana* cells

**Fig 1:**
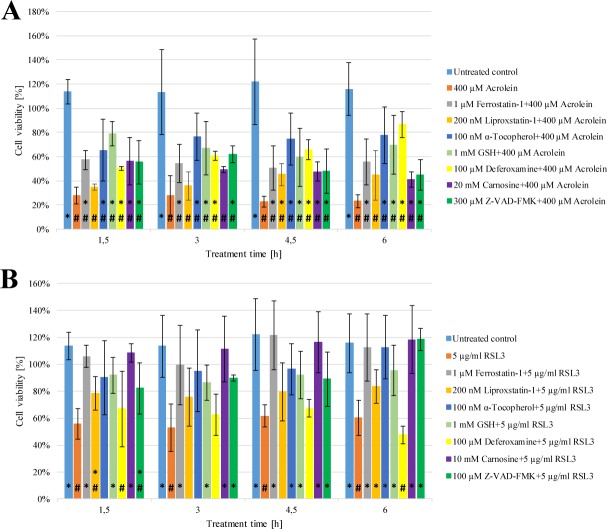
**Comparison of the cytotoxic effect of acrolein (Panel A) and RSL3 (Panel B) on *Arabidopsis thaliana* cells.** *A*. *thaliana* suspension cells were pre-treated with either Ferrostatin-1 (grey), Liproxstatin-1 (gold), α-Tocopherol (dark blue), GSH (light green), Deferoxamine (yellow), Carnosine (purple) or Z-VAD-FMK (dark green) for 1 h. Subsequently the cells were treated with 400 μM Acrolein (orange, Panel **A**) or 11.34 μM (5 μg/ml) RSL3 (orange, Panel **B**). At the indicated time points, the viability of the cells was determined by TTC reduction assay as described in Materials and methods. The cell viability right before the addition of Acrolein (**A**) or RSL3 (**B**) was regarded as 100%. The data shown are means ± SD from at least three different treatments. * represents significant differences with respect to “400 μM Acrolein” (**A**) or “5 μg/ml RSL3” (**B**). # represents significant differences with respect to untreated control (**A**, **B**) (p<0.05).

Acrolein induced cell death and heat treatment induced FCD in *Arabidopsis thaliana* share common features such as production of ROS, lipid peroxidation, GSH and ascorbate depletion [10,15]. Thus, in the first set of our experiments we aimed at the investigation of the possible involvement of FCD in acrolein induced cell death and the possible involvement of acrolein in the ferroptosis-like pathway. The cells were treated with acrolein or with the known ferroptosis inducer RSL3 in the absence and in the presence of different ferroptosis inhibitors (Ferrostatin-1, Liproxstatin-1, α-Tocopherol, GSH, Deferoxamine) and the cell viability was monitored for 6 h. As quick as 1.5 h after the addition of acrolein, the cell viability decreased to 25% of the control, and did not recover in the whole investigated time interval (Fig 1 Panel A). Although RSL3 caused a slightly lower initial cell death rate (~50%) the cell viability pattern of both cell death inducers were quite similar in the investigated period (Fig 1 Panel A and B). The cytotoxic effect of acrolein could be mitigated by pre-treatment of the cells with known ferroptosis inhibitors such as Ferrostatin-1, Deferoxamine, α-Tocopherol, and GSH (Fig 1 Panel A). However, none of the ferroptosis inhibitors could elevate the cell viability to the level of the untreated control (Fig 1 Panel A). Glutathione and α-Tocopherol were the most potent inhibitors, while Ferrostatin-1 had the weakest, but still significant positive effect (Fig 1 Panel A). The addition of Liproxstatin-1 had a slightly positive, but non-significant effect on cell viability (Fig 1 Panel A). Ferrostatin-1 and GSH could protect the cells against RSL3 induced cell death (Fig 1 Panel B). At shorter incubation times Liproxstatin-1 and α-Tocopherol had non-significant positive effect on the viability of RSL3 treated cells, however at longer incubation times this effect became significant (Fig 1 Panel B). Interestingly, Deferoxamine elevated the cell viability of acrolein treated cells clearly, but had no positive effect on the viability of RSL3 treated cells (Fig 1 Panel A and B).

Since acrolein is a lipid peroxide-derived reactive carbonyl species the effect of the reactive carbonyl species scavenger dipeptide, Carnosine was also investigated in both acrolein and RSL3 treated cells. It could moderately elevate the cell viability in acrolein treated and more significantly in RSL3 treated cells (Fig 1 Panel A and B). Acrolein is also a known activator of caspase-1-like and caspase-3-like proteases [15,21] thus the effect of Z-VAD-FMK was also investigated on cell viability. Surprisingly the observed fall of cell viability due to acrolein treatment was only mitigated, but the reduction of cell viability due to RSL3 treatment was totally abolished by Z-VAD-FMK (Fig 1 Panel A and B).

### The effect of acrolein and RSL3 treatment on the generation of ROS

**Fig 2:**
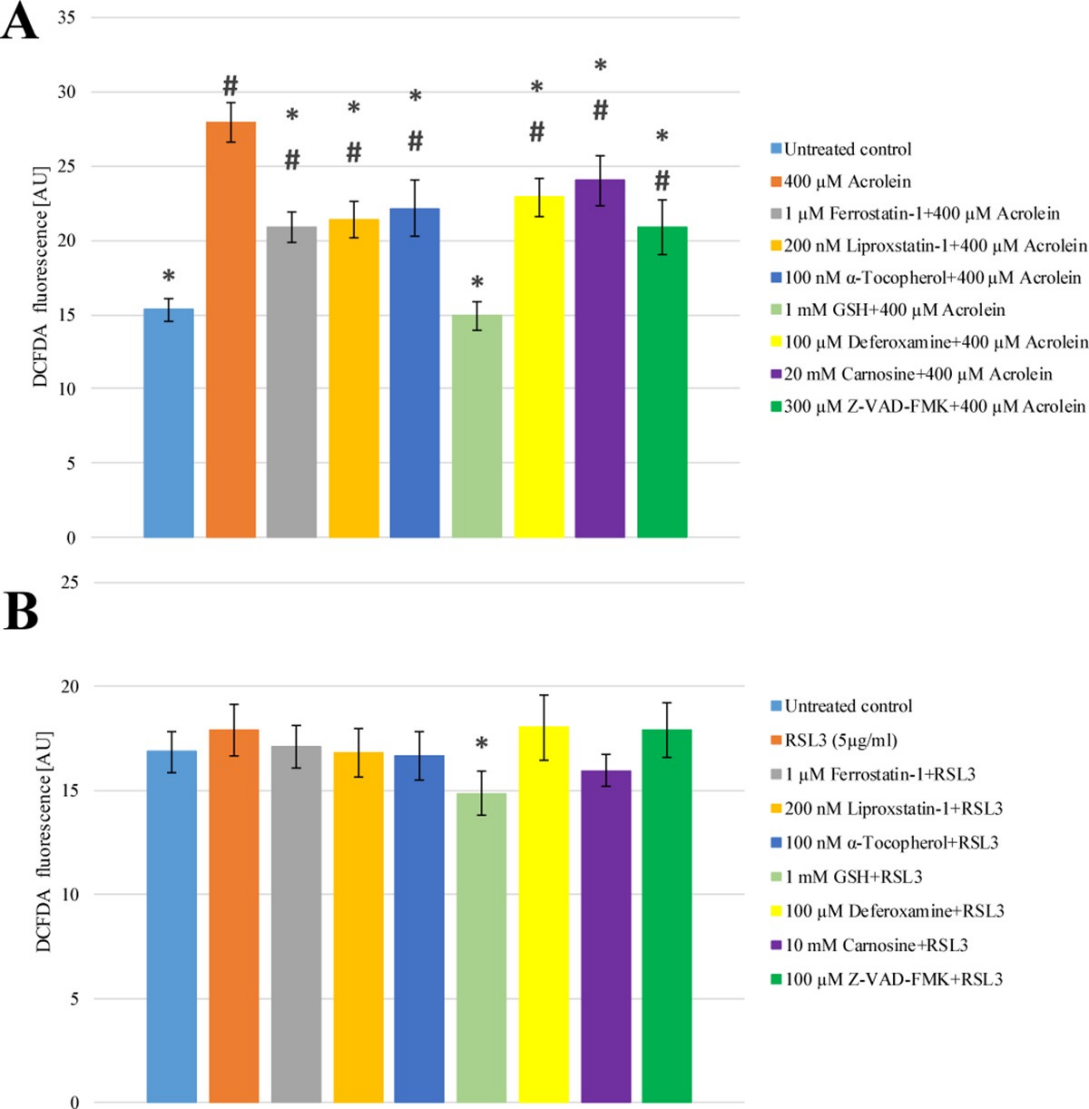
**The effect of acrolein (Panel A) and RSL3 (Panel B) treatment on the generation of ROS in *Arabidopsis thaliana* suspension cells.** *A*. *thaliana* suspension cells were pre-treated with either Ferrostatin-1 (grey), Liproxstatin-1 (gold), α-Tocopherol (dark blue), GSH (light green), Deferoxamine (yellow), Carnosine (purple) or Z-VAD-FMK (dark green) for 1 h. Subsequently the cells were treated with 400 μM Acrolein (orange, Panel **A**) or 5 μg/ml RSL3 (orange, Panel **B**) for 3 h. ROS formation was determined by H_2_DCFDA assay as described in Materials and Methods. The data shown are means ± SD from at least three different treatments. * represents significant differences with respect to “400 μM Acrolein” (**A**) or “5 μg/ml RSL3” (**B**). # represents significant differences with respect to untreated control (**A**, **B**) (p<0.05).

**Fig 3:**
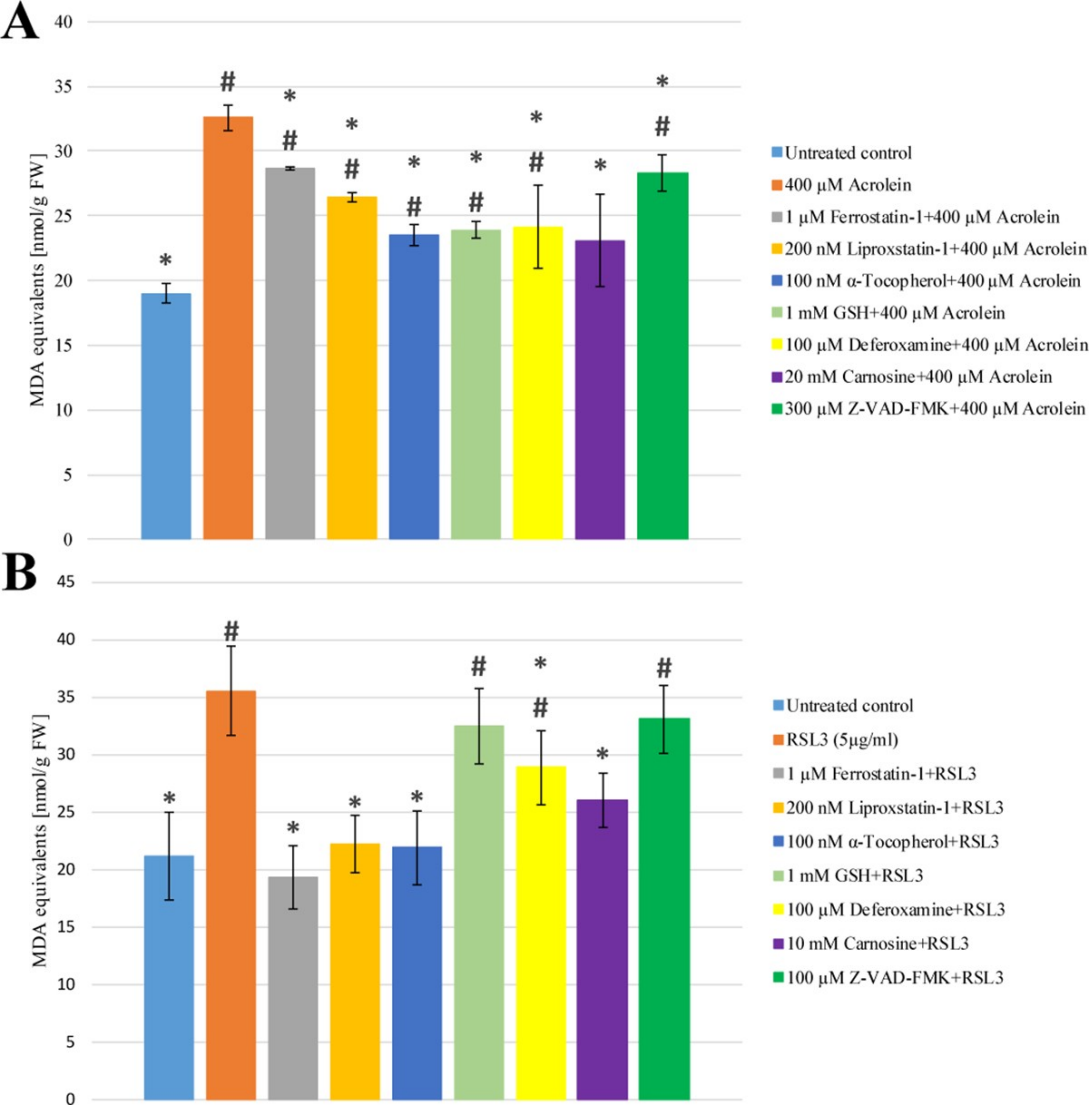
**The effect of acrolein (Panel A) and RSL3 (Panel B) treatment on the generation of lipid peroxides in *Arabidopsis thaliana* suspension cells.** *A*. *thaliana* suspension cells were pre-treated with either Ferrostatin-1 (grey), Liproxstatin-1 (gold), α-Tocopherol (dark blue), GSH (light green), Deferoxamine (yellow), Carnosine (purple) or Z-VAD-FMK (dark green) for 1 h. Subsequently the cells were treated with 400 μM Acrolein (orange, Panel **A**) or 5 μg/ml RSL3 (orange, Panel **B**) for 3 h. Lipid peroxidation was estimated by TBARS assay as described in Materials and Methods. The data shown are means ± SD from at least three different treatments. * represents significant differences with respect to “400 μM Acrolein” (**A**) or “5 μg/ml RSL3” (**B**). # represents significant differences with respect to untreated control (**A**, **B**) (p<0.05).

The generation of ROS, especially lipid ROS plays crucial role in the initiation of ferroptosis [6,10], thus their generation due to RSL3 and acrolein treatment was followed. Both H_2_DCFDA detectable and lipid ROS generation was significantly increased by acrolein treatment (Figs 2 and 3 Panel A). Both of them could be significantly mitigated by pre-treating the cells with Ferrostatin-1, Liproxstatin-1, α-Tocopherol, GSH, Deferoxamine, Carnosine or Z-VAD-FMK (Figs 2 and 3 Panel A). Interestingly, no similar elevation of ROS generation could be observed due to RSL3 treatment (Fig 2 Panel B). However, the level of lipid ROS was enhanced as significant as in acrolein treatment (Fig 3 Panel B). Ferrostatin-1, Liproxstatin-1, α-Tocopherol and Carnosine could fully prevent the RSL3 induced lipid peroxidation. Deferoxamine also showed significant protective effect, however it could not decrease the level of lipid peroxidation to that of the untreated control (Fig 3 Panel B).

### The effect of acrolein and RSL3 treatment on the cellular GSH level

**Fig 4:**
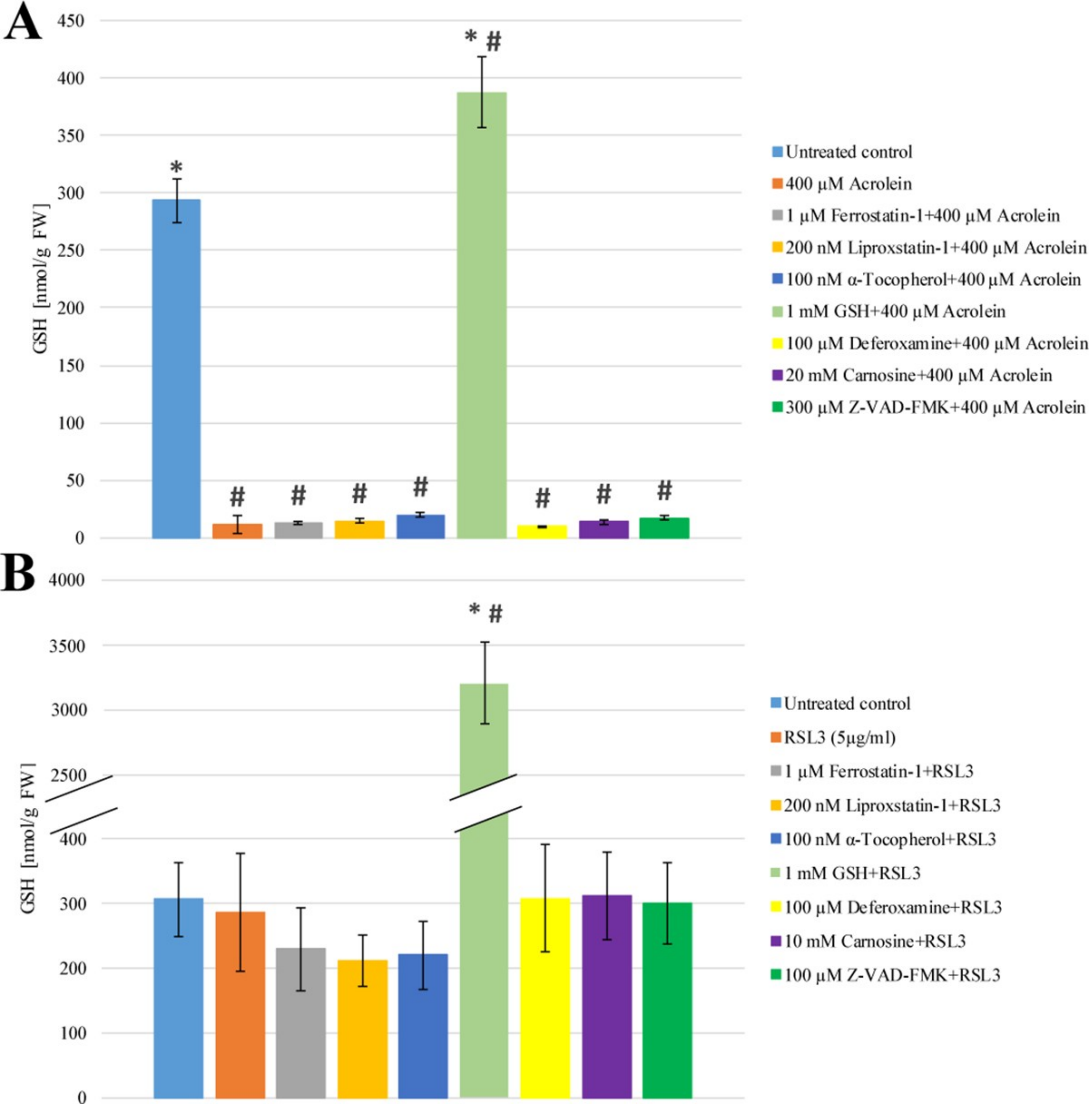
**The effect of acrolein (Panel A) and RSL3 (Panel B) treatment on the level of GSH in *Arabidopsis thaliana* suspension cells.** *A*. *thaliana* suspension cells were pre-treated with either Ferrostatin-1 (grey), Liproxstatin-1 (gold), α-Tocopherol (dark blue), GSH (light green), Deferoxamine (yellow), Carnosine (purple) or Z-VAD-FMK (dark green) for 1 h. Subsequently the cells were treated with 400 μM Acrolein (orange, Panel **A**) or 5 μg/ml RSL3 (orange, Panel **B**) for 3 h. GSH content was determined by DTNB assay as described in Materials and Methods. The data shown are means ± SD from at least three different treatments. * represents significant differences with respect to “400 μM Acrolein” (**A**) or “5 μg/ml RSL3” (**B**). # represents significant differences with respect to untreated control (**A**, **B**) (p<0.05).

The level and redox status of GSH reflect to the extent of oxidative stress and play critical role in the development of ferroptosis, thus it was also investigated in both RSL3 and acrolein treated cells. As it was expected, the cellular glutathione pool was significantly depleted due to acrolein treatment, while RSL3 did not cause any significant changes in it (Fig 4 Panel A and B). Although the extent of lipid peroxidation could be significantly lowered by Ferrostatin-1, Liproxstatin-1, α-Tocopherol, GSH, Deferoxamine, Carnosine or Z-VAD-FMK pre-treatment of the cells, none of these inhibitors could prevent the depletion of the GSH pool (Fig 4 Panel A).

### The effect of acrolein and RSL3 treatment on the activity of caspase-3- like protease

**Fig 5:**
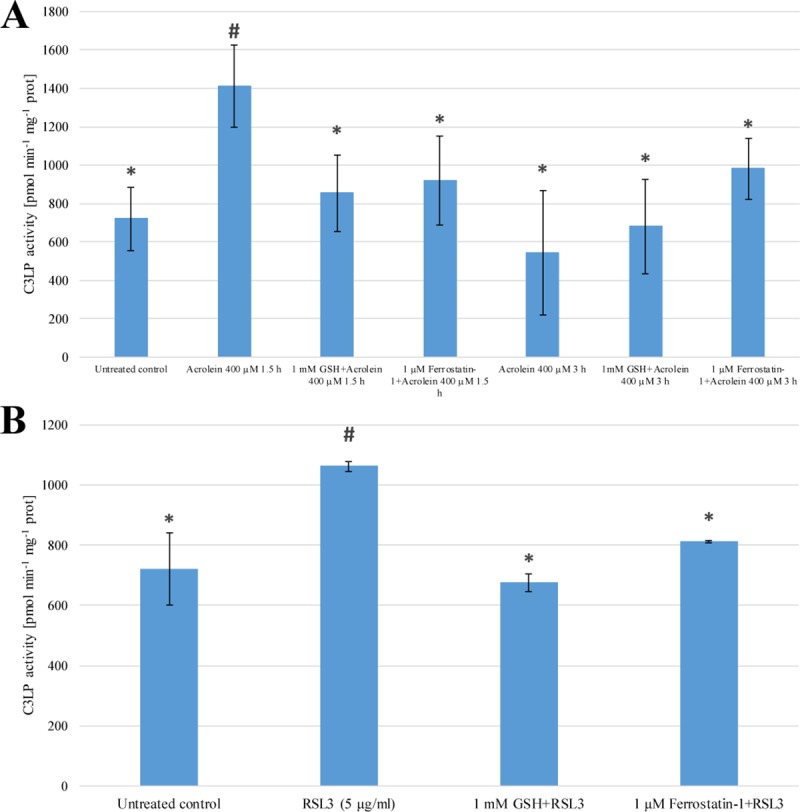
**The effect of acrolein (Panel A) and RSL3 (Panel B) treatment on the activity of caspase-3-like protease of *Arabidopsis thaliana* suspension cells.** *A*. *thaliana* suspension cells were pre-treated with either 1μM Ferrostatin-1, or 1 mM GSH for 1 h. Subsequently the cells were treated with 400 μM Acrolein (Panel **A**) or 5 μg/ml RSL3 (Panel **B**) for 3 h. The C3LP activity was determined by the measurement of AMC fluorescence as described in Materials and Methods. The data shown are means ± SD from at least three different treatments. * represents significant differences with respect to “400 μM Acrolein 1.5 h” (**A**) or “5 μg/ml RSL3” (**B**). # represents significant differences with respect to untreated control (**A**, **B**) (p<0.05).

On one hand acrolein is a known activator of caspase-3-like proteases, on the other hand Z-VAD-FMK behaved as a potent inhibitor of both acrolein and RSL3 induced cell death, thus the effect of RSL3 (together with acrolein) on the activity of caspase-3-like protease was investigated. Elevated caspase-3-like protease activity could be measured due to acrolein treatment (Fig 5 Panel A). RSL3 treatment of the cells also resulted in enhanced caspase-3-like protease activity (Fig 5 Panel B). The observed elevation of caspase-3-like protease activity could be significantly mitigated by GSH or Ferrostatin-1 pre-treatment of the cells (Fig 5 Panel A and B). GSH showed more significant protection against the activation of caspase-3-like protease in both cases (Fig 5 Panel A and B). Since acrolein caused the rapid decrease of cell viability (Fig 1 Panel A) the activity of caspase-3-like protease due to acrolein treatment was given at both 1.5 and 3 h of incubation times.

## Discussion

Ferroptosis, the iron dependent cell death, was originally described in RAS mutant tumour cell lines [6,22,23]. In addition its presence in plants was also described recently [10]. Similar to the human cells, heat stress induced FCD in plant cells was accompanied by the accumulation of lipid ROS, and depletion of GSH [10]. The cell death showed iron dependency and could be prevented by the canonical ferroptosis inhibitors, Ferrostatin-1 and the iron chelator ciclopirox olamine [10]. The overexpression of enzymes responsible for detoxification of toxic lipid metabolites leads to ferroptosis resistance supporting the possible role of these reactive aldehydes in ferroptotic cell death [9]. It was found that acrolein, one of the lipid peroxide derived reactive carbonyl species, generated under environmental stress caused the depletion of the GSH pool in BY-2 tobacco cells. This effect gradually lowered the ascorbate level and enhanced the ROS level in the cell, followed by cell death at the end [15]. All these observations are substantially similar to the results found in the case of heat treatment induced FCD in *Arabidopsis thaliana* [10]. The similar features of acrolein induced cell death and heat stress induced ferroptosis raised the hypothesis that this molecule can be another ferroptosis inducer. Furthermore, it might also be a good candidate as a ferroptotic mediator.

Thus, the effect of both the known ferroptosis inductor RSL3 and acrolein was investigated on the cell viability of *Arabidopsis thaliana* to elucidate the possible involvement of ferroptosis in acrolein induced cell death and the possible involvement of acrolein in the ferroptotic pathway. Both compounds caused the significant decrease of cell viability (Fig 1 Panel A and B). The cytotoxic effect of acrolein could be mitigated by pre-treatment of the cells with well-known ferroptosis inhibitors such as Ferrostatin-1, Deferoxamine, α-Tocopherol, and GSH (Fig 1 Panel A). This observation clearly indicates that ferroptosis is involved in acrolein induced cell death. The similar inhibitory profile of the known ferroptosis inducer RSL3 (Fig 1 Panel A and B) further confirmed that ferroptosis is, at least partly, responsible for the acrolein induced cell death in plant cells. Interestingly, the cell permeable iron chelator Deferoxamine elevated the cell viability of acrolein treated cells clearly, but positive effect on the viability of RSL3 treated cells was not observed (Fig 1 Panel A and B). The different effect of Deferoxamine on various ferroptosis inductors is not without any example since similar effect could be observed in RSL3 and erastin treated acute lymphoblastic leukemia (ALL) cells [24]. Furthermore, different anti-ferroptotic effect of Deferoxamine could also be observed on distinct human cancer cell lines [25]. The different effect of Deferoxamine on cells treated by various ferroptosis inducers or on distinct cell lines treated by the same ferroptosis inducers showed its non-uniform behaviour. Unfortunately, there has not been taken any experimental comparison in the case of plant cells treated by different ferroptosis inducers and Deferoxamine.

The reactive carbonyl species scavenger dipeptide, Carnosine could successfully scavenge acrolein and mitigate cell death earlier [15,21]. In our experiments it could also moderately elevate the cell viability in acrolein treated cells and even more significantly in RSL3 treated cells (Fig 1 Panel A and B). According to these results we suppose that both cell death inducers can act on a similar way. The protective effect of Carnosine in RSL3 treated cells also raised the possible involvement of reactive carbonyl species (acrolein) in the RSL3 induced (ferroptosis-like) cell death in plant cells.

Acrolein is also a well-known activator of both caspase-1-like and caspase-3-like proteases [15,21] therefore the effect of the cell-permeable caspase inhibitor Z-VAD-FMK was also investigated on cell viability. It is worth to note that Z-VAD-FMK had similar inhibitory effect on the heat stress induced plant cell death [26]. Surprisingly the observed decrease of cell viability due to acrolein treatment was only mitigated, but the reduction of cell viability due to RSL3 treatment was totally abolished by Z-VAD-FMK (Fig 1 Panel A and B). Our new results are in concordance with the results of Distefano *et al*. [10] who found that heat stress induced FCD could be mitigated by the inhibition of caspase-like activity. The results of Mano *et al*. [15] are reinforced by the elevated caspase-3-like protease activity due to acrolein treatment (Fig 5 Panel A). RSL3 treatment also resulted in enhanced caspase-3-like protease activity, that could be significantly mitigated by GSH or Ferrostatin-1 pre-treatment (Fig 5 Panel A and B). All these observations demonstrate, that caspase-like activity is clearly involved in RSL3 (Fig 1 Panel A and B) and heat stress [10] induced FCD in plant cells.

ROS generation, especially lipid ROS production play crucial role in the initiation of ferroptosis [6,10]. Therefore, the elucidation of the detailed mechanism of RSL3 induced cell death and the clarification of the relationship of acrolein and ferroptosis in plant cells could only be carried out by the investigation of ROS generation due to RSL3 and acrolein treatment. The H_2_DCFDA detectable ROS formation was significantly increased by addition of acrolein. Parallel, enhanced lipid peroxidation could also be observed (Figs 2 and 3 Panel A), but no similar elevation of H_2_DCFDA detectable ROS generation could be observed due to RSL3 treatment (Fig 2 Panel B). However, the level of lipid ROS was enhanced as significantly as in acrolein treatment (Fig 3 Panel B). Both level and redox status of GSH are excellent markers of oxidative stress [27]. Furthermore, the level of GSH also plays crucial role in the development of ferroptosis [6,10], thus it was also followed due to RSL3 and acrolein treatments. According to the earlier observation of Mano and Biswas [15], the cellular GSH pool was significantly depleted due to acrolein treatment, which could not be prevented by the addition of any ferroptosis inhibitor (Fig 4 Panel A). It is in concordance with the earlier observations of our and other research groups, since these inhibitors do not act through the elevation or stabilization of the GSH level [28,29]. RSL3, as an inhibitor of GPX4, inhibits the elimination of lipid peroxides and has no effect on the level of GSH [30], accordingly it did not cause any significant changes in the level of GSH (Fig 4 Panel B) and it was not involved in the generation of ROS, while acrolein treatment was accompanied by the generation of ROS [15]. It was clearly demonstrated that GSH is an extraordinary efficient anti-cell death agent (Fig 1) and it also gave good protection against both the lipid and non-lipid ROS (Figs 2 and 3). RSL3 is a selective GPX inhibitor that has much more focused effect than acrolein [30]. Thus, it is possible that the lower cellular GSH content can be in the background of the observed lower efficiency of Carnosine protection against direct acrolein treatment (Fig 1 Panel A). Similarly, the observed higher H_2_DCFDA detectable ROS formation due to acrolein treatment can also be described by the different action of acrolein and RSL3 (Fig 2). Acrolein induced ROS generation and lipid ROS formation could be significantly mitigated by pre-treating the cells with ferroptosis inhibitors, the acrolein scavenger Carnosine and the cell-permeable caspase inhibitor Z-VAD-FMK (Figs 2 and 3 Panel A). These observations further strengthen the role of FCD in acrolein induced cytotoxicity and the possible role of caspase-like proteases in FCD. Therefore, on the contrary to the caspase independent ferroptosis in human cells [6] it is found that caspase-like activity can be involved in plant FCD. Necessarily there is a long way to go and more experiments have to be performed to elucidate the details of FCD in plant cells and to clarify the similarities and differences of animal and plant cell death.

## Supporting information

S1 File Data set of all experiments. (XLSX)
